# Internalizing and Externalizing Traits During Adolescence: Using Epigenetics and Perinatal Risks to Differentiate Clusters of Symptoms

**DOI:** 10.3390/biom15081142

**Published:** 2025-08-07

**Authors:** Maddalena Mauri, Silvia Grazioli, Carolina Bonivento, Alessandro Crippa, Roberto Giorda, Eleonora Maggioni, Fabiana Mambretti, Eleonora Rosi, Letizia Squarcina, Federica Tizzoni, Paolo Brambilla, Maria Nobile

**Affiliations:** 1 Child Psychopathology Unit, Scientific Institute, IRCCS Eugenio Medea, 23842 Bosisio Parini, Italy; maddalena.mauri@lanostrafamiglia.it (M.M.); alessandro.crippa@lanostrafamiglia.it (A.C.); eleonora.rosi@lanostrafamiglia.it (E.R.); federica.tizzoni@lanostrafamiglia.it (F.T.); 2 Department of Psychology, Sigmund Freud University, 20143 Milan, Italy; s.grazioli@milano-sfu.it; 3 Studi Cognitivi, Cognitive Psychotherapy School and Research Centre, 20143 Milan, Italy; 4 Scientific Institute, IRCCS Eugenio Medea, 33037 Pasian di Prato, Italy; carolina.bonivento@lanostrafamiglia.it; 5 Laboratory of Medical Genetics, Scientific Institute, IRCCS Eugenio Medea, 23842 Bosisio Parini, Italyfabiana.mambretti@lanostrafamiglia.it (F.M.); 6 Department of Electronics, Information and Bioengineering (DEIB), Politecnico di Milano, 20133 Milan, Italy; eleonora.maggioni@polimi.it; 7 Department of Neurosciences and Mental Health, Fondazione IRCCS Ca’ Granda Ospedale Maggiore Policlinico, 20122 Milan, Italy; letizia.squarcina@unimi.it (L.S.); paolo.brambilla1@unimi.it (P.B.); 8 Department of Pathophysiology and Transplantation, University of Milan, 20122 Milan, Italy

**Keywords:** clustering, DNA methylation, adverse life events, psychopathological traits, adolescence

## Abstract

This cross-sectional study aims to identify clusters of internalizing and externalizing traits during adolescence using a bottom-up approach. The second aim is to investigate whether the different clusters differ by environmental risk factors and specific epigenetic profiles. A total of 205 adolescents, who had been referred for psychopathology in childhood, were recruited. Behavioral problems were assessed using the Child Behavior Checklist/6–18 (CBCL). Different clusters of psychopathological profiles were analyzed using a Finite mixture model. Differences in environmental risk factors and epigenetic profiles were tested with χ^2^-tests and Bonferroni-corrected t-tests. Two clusters were identified: a LOW cluster (51% of the sample), characterized by the presence of subclinical mean scores in both internalizing and externalizing problems, and a HIGH cluster (49% of the sample), characterized by high mean scores in both domains. The HIGH cluster had a significantly greater number of perinatal complications and changes in methylation of specific CpG sites of *Brain-derived neurotrophic factor*, *Insulin-like growth factor-2*, and *Oxytocin receptor*, whereas no difference was found for *FK506-binding protein 5*. Our results confirm the existence of a strong association between early adverse events, DNA methylation, and the presence of behavioral problems and psychopathological traits in adolescence.

## 1. Introduction

Adolescence is a challenging period in which an individual is confronted with various changes related to biological development and environmental requests [1,2,3,4]. This process often leads to a decrease in psychological well-being. Previous evidence indicated that 18% of Italian adolescents have some form of emotional and/or behavioral problems [1,2,3], which can persist into adulthood [5]. To better understand the complexity of psychopathology, recent research has focused on the description of psychopathological traits, such as internalizing and externalizing difficulties, rather than the presence/absence of a categorical psychiatric diagnosis [6]. This approach has been shown to be effective in identifying different groups of adolescents with different combinations of internalizing and externalizing problems, which could lead to better tailored prevention and intervention programs [5,7].

In developmental psychopathology, the use of the terms “internalizing” and “externalizing” traits refers to transdiagnostic behaviors: internalizing problems are inwardly directed negative behaviors (e.g., anxiety, depression, and somatic symptoms), whereas externalizing problems refer to outwardly directed negative behaviors (e.g., hyperactivity, aggression, disruptive behavior, and substance use) [8,9].

When approaching psychopathology, it is also important to consider environmental (risk) factors to understand the heterogeneous presentation of symptoms over the course of development [10,11,12]. Previous works have shown that both early negative experiences in the pre- and perinatal period [9,12,13,14,15] and later stressful experiences [16] are highly associated with long-term negative consequences for the individual.

With this respect, epigenetics can explain the mechanism underlying the development of psychopathology due to unfavorable environmental events [17]. Epigenetic events are modifications of DNA, RNA, or the structural regulatory proteins bound to them that alter gene transcription and protein production without altering nucleotide sequences [18]. The presence of altered methylation profiles as a result of environmental influences has been demonstrated in animal and human studies [19,20,21].

Several studies (e.g., [18,22,23,24,25]) documented changes in DNA methylation of various genes in individuals exposed to stressful negative experiences during prenatal (e.g., maternal depression) and postnatal (e.g., childhood abuse) life. These studies focused on methylation processes involving genes associated with the development of psychopathological traits in response to early stress and trauma, such as *Brain-derived neurotrophic factor* (*BDNF*), *FK506-binding protein 5* (*FKBP5*), *Insulin-like growth factor-2* (*IGF2*), and *Oxytocin receptor* (*OXTR*) [25,26,27,28,29,30].

To date, there are no studies linking different profiles of internalizing and externalizing traits in adolescence to early adverse experiences and epigenetic risk factors.

In this study, we investigated whether the formation of subgroups of adolescents based on their internalizing and externalizing traits could provide a more accurate way to differentiate how different early environmental and epigenetic events influence them.

First, we used an unbiased, data-driven approach to identify different groups of adolescents by analyzing externalizing and internalizing problems on the CBCL/6-18 [8].

Second, we tested whether the identified clusters of adolescents differed by the presence of environmental risk factors (i.e., the presence of stressful life events, pre- and postnatal risk factors, socioeconomic and demographic characteristics) and specific epigenetic profiles (specifically, DNA methylation in specific portions of four candidate genes, i.e., *BDNF*, *FKBP5*, *IGF2*, and *OXTR*). For *BDNF*, the region in intron 1 of the gene was selected as previous studies have shown altered methylation of *BDNF* in response to unfavorable living conditions [31,32,33]. For *OXTR*, three genomic regions were investigated: (i) a region within intron 1 that has previously been associated with autism symptoms and both externalizing and internalizing behaviors [32,34,35]; (ii) the promoter region upstream of the start codon, which plays a crucial role in the control of *OXTR* transcription [35]; and (iii) a region within exon 3 that has been associated with various psychopathologies, including anxiety and depression, obsessive–compulsive disorder, post-traumatic stress disorder, and deficits in social communication [36,37]. In addition, hypermethylation at CpG sites in this region has already been associated with poor maternal care in childhood [29]. In *IGF2*, the analyzed region is one of the differentially methylated regions (DMRs) that contribute to the regulation of *IGF2*, an imprinted gene expressed from the paternally derived chromosome [38]. Alterations in the methylation of this DMR have been associated with exposure to prenatal risk factors such as caloric restriction in utero [39] and cigarette smoking [40]. The analyzed CpG sites within *FKBP5* are located in a regulatory region of intron 7, which is particularly important for cellular differentiation and proliferation [41]. The altered methylation of these CpG sites has previously been associated with neurobehavioral outcomes [42].

Based on previous findings, we hypothesized that we would find 2–4 clusters of participants characterized by different profiles of internalizing and externalizing traits. We also expected that subgroups with greater emotional/behavioral difficulties would have an increased likelihood of environmental and epigenetic risk factors.

## 2. Materials and Methods

### 2.1. Study Design

The present work is an observational study with a cross-sectional character. In particular, the data presented here were collected during the follow-up of a longitudinal project [43,44,45,46,47] at prepubertal age. The study protocols were approved by the Ethical Research Committee of the IRCCS Eugenio Medea Scientific Institute (protocol code: Prot.N. 022/12-CE) and were conducted in accordance with the ethical standards of the 1964 Declaration of Helsinki and its subsequent amendments. Written parental consent was obtained for all participants.

### 2.2. Sample

The study sample consisted of 205 adolescents recruited in Northern Italy. All participants were referred to the Eugenio Medea Scientific Institute in childhood (mean age 8.56 ± 1.96 years) for emotional and behavioral problems and were re-examined at the 5-year follow-up [43,44,45,46,47]. Exclusion criteria were diagnoses of autism spectrum disorder or intellectual disability, neurological disorders (including epilepsy and traumatic brain injury), and severe sensory and language comprehension deficits.

Neuropsychiatric diagnoses at initial screening were 43.90% anxiety disorders, 31.71% attention-deficit/hyperactivity disorder, 12.20% mood disorders, 4.88% oppositional defiant disorder or conduct disorder, one respondent had obsessive–compulsive disorder, and 6.83% of the sample did not receive a categorical diagnosis. Categorical diagnoses were not recorded during adolescence.

### 2.3. Measures and Variables

#### 2.3.1. Clinical Information

Behavioral problems were assessed using the CBCL [8] from the Achenbach System of Empirically Based Assessment (ASEBA), which was completed by the parents. This questionnaire can be used to assess a broad spectrum of emotional, behavioral, and social problems in children and adolescents. It provides 8 empirically based syndromal scales and 3 summary scales based on the syndromic scales (i.e., the Total Problems scale, the Internalizing Problems scale, which includes the Anxious/Depressed, Withdrawn/Depressed and Somatic Complaints scales, and the Externalizing Problems scale, which includes the Rule Breaking and Aggressive Behaviour scales). According to the Manual for the ASEBA school-age forms and profiles [8], for the Internalizing and Externalizing scales, T-scores < 60 are considered in the normal range, T-scores ≥ 60 and <64 are considered borderline, and T-scores ≥ 64 are considered in the clinical range.

The T-scores of the Internalizing and Externalizing scales were used as input variables for the development of the cluster algorithm.

#### 2.3.2. Environmental Risk Factors

Stressful life events (SLEs): The presence of SLEs was assessed using parental reports with the Development And Well-Being Assessment—DAWBA interview [48]. The following SLEs were listed in the background section of the interview: a serious accident or serious injury in an accident; a serious illness requiring hospitalization; the death of a parent, sibling, or close friend; the end of a close friendship; a major financial crisis in the family (e.g., the loss of the equivalent of three months’ income); other stressful events affecting the child or family. The presence of SLEs (no SLE vs. one or more SLEs) was considered a categorical dependent variable.

Perinatal risk factors: The presence of pre- and perinatal risk factors was assessed using an ad hoc questionnaire completed by the parents. The questionnaire asked about the presence of the following prenatal risks: viral, parasitic, or bacterial infections during pregnancy (i.e., rubella, syphilis, influenza, rubella, Toxoplasma gondii, herpes simplex virus—type 2, Borna disease), RH factor incompatibility, pre-eclampsia, and threatened termination of pregnancy. Perinatally assessed risks were premature rupture of membranes, labor > 24 h or “rushed”, twin birth—single or complicated, prolapse or rupture of the umbilical cord or kinking of the umbilical cord around the newborn’s neck, premature birth with a gestational length < 37 weeks or delayed delivery with duration of gestation > 42 weeks, cesarean section—complicated or urgent, breech or abnormal position, use of forceps or other delivery instruments, birth weight < 2 kg, incubator/resuscitation/“blue baby”, and newborn with gross physical abnormalities. The presence of pre- or perinatal risks (no pre- and/or perinatal risk vs. one or more pre- and/or perinatal risks) was considered a categorical dependent variable.

#### 2.3.3. Epigenetic Measures

DNA methylation levels were measured from saliva samples collected using non-invasive methods, to increase patients’ compliance. Moreover, saliva samples contain cells derived from ectoderm (like the brain), whereas blood derives from mesoderm. Before starting the assessment, saliva samples were collected from the participants approximately at 9.00 AM after a minimum 1 h fast(see Appendix A for details of sample collection and genetic analysis; Appendix B for distribution of methylation in *BDNF*, *FKBP5*, *IGF2*, and *OXTR* portions).

The methylation status of specific portions of the *BDNF*, *FKBP5*, *IGF2*, and *OXTR* genes was analyzed by PCR amplification of bisulfite-treated DNA followed by next-generation sequencing (NGS). To measure the degree of bisulfite conversion, we verified that CT conversion was greater than 99% for non-CpG cytosines. As a control, we included in our study a sample, previously analyzed with an independent experiment, for which the methylation degree at certain sites was known.

In detail, we analyzed the following regions (human genome assembly GRCh37/hg19):

• *A region within intron 1 of BDNF* (chr11:27723077–27723244, 11 CpGs).
• *A region within intron 7 of FKBP5* (chr6:35558405–35558550, 3 CpGs).
• *IGF2* differentially methylated region (DMR, chr11:2169373–2169658, 5 CpGs).
• Three regions of *OXTR*: one in the promoter (chr3:8811488–8811837, 7 of 9 CpGs analyzed), one in intron 1 (chr3:8810654–8810919, 13 CpGs), and one in exon 3 (chr3:8809340–8809530, 15 CpGs).

Paired-end reads from each sample were independently aligned to all reference sequences using a parallel Smith–Waterman algorithm [49]. Only paired-end reads that aligned coherently to the same reference sequence were retained. At each CpG site in each sequence, the 4 base frequencies were analyzed and reported together with the C→T percentage. Samples that did not have a coverage of at least 100x in each CpG analyzed were excluded.

Methylation data, grouped into percentages and reads, were screened and trimmed based on the number of reads for each subject and portion of the genes. The percentage of methylation in each portion of the genes was considered a continuous dependent variable.

### 2.4. Data Analysis

#### 2.4.1. Preliminary Procedures on Data and Descriptive Statistics

Statistical analyses were performed using the statistical software R (R core Team, 2021, version 4.1.0). Data cleaning procedures were performed according to the manual by Van der Loo and De Jonge [50]; specifically, participants with more than 50% missing data were removed and the remaining missing data were imputed by random imputation using the Hmisc R package [51]. Random imputation is a stochastic method for handling missing data in which each missing value is replaced by a randomly selected observed value of the same variable. This approach preserves the empirical distribution and variance of the observed data, thereby maintaining the distributional properties of the original variables. The distribution of the continuous variables in the entire sample was then checked and descriptive statistics were calculated.

#### 2.4.2. Unsupervised Machine Learning: Cluster Analysis on Clinical Measures

The presence of homogeneous clusters of psychopathological traits was assessed using an unsupervised algorithm, the Finite mixture model (FMM), using the R package “mclust” [52,53]. The FMM was implemented on the T-scores of the Internalizing and Externalizing scales of the CBCL [8]. Models estimating solutions of two or more clusters were compared using the Bayesian Information Criterion (BIC), and the top three models identified were EII with 2 clusters, EII with 3 clusters, and VII with 2 clusters. Among these, the optimal solution was the EEI model with 2 clusters, which assumes a diagonal covariance structure with equal shape across clusters. Therefore, EEI and two clusters were used as the final model parameters. Table A2 in Appendix B reports the Bayesian Information Criterion (BIC) values.

#### 2.4.3. Analysis of Cluster Characteristics

χ^2^ were conducted to analyze possible differences between clusters in the presence of environmental risk as a categorical variable; Bonferroni-corrected *t*-tests were conducted to analyze possible differences between clusters in the CBCL scales for behavioral problems and sociodemographic and methylation characteristics. The alpha level for these analyses was corrected with the Bonferroni correction for multiple comparisons.

## 3. Results

After removing participants with more than 50% missing data, 200 participants (76% males, age 14.45 ± 2.16 years) remained for the analyses. The mean SES was 48.95 ± 19.39 (range: 0–90). The sample was characterized by the presence of internalizing and externalizing behavioral problems within the cutoffs of normality (Internalizing scale T-scores mean: 57.1 ± 9.02, range: 34–78; Externalizing scale T-scores mean: 53.2 ± 9.05, range 34–86). Regarding environmental risk factors, SLEs were present in 42% of the sample (range: 0–4) and perinatal risk factors were present in 44% (range: 0–8). The characteristics of the methylation variables are shown in Table 1.

### 3.1. Unsupervised Machine Learning Results: Results of Cluster Analysis on Clinical Measures

**Figure 1:**
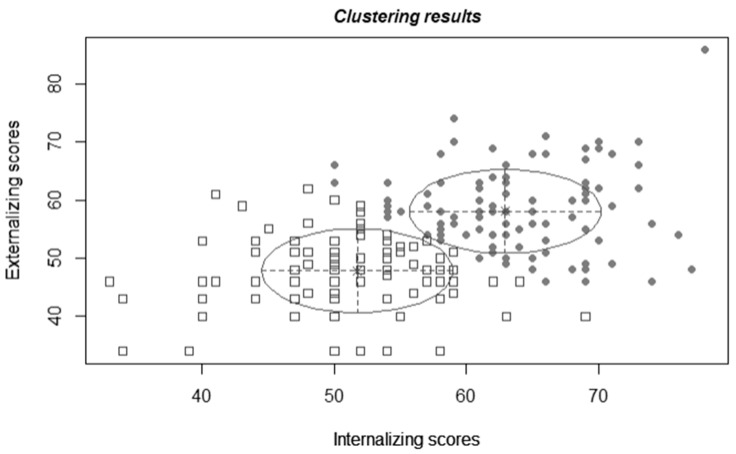
Cluster analysis. Notes: The squares represent participants belonging to the “LOW” cluster, while circles represent participants belonging to the “HIGH” cluster. The values for internalizing and externalizing behaviors are expressed in T-scores, which have a mean of 50 and a standard deviation of 10.

The results of the analysis are shown in Figure 1. The best selected model (BIC = −2887.49, log-likelihood = −1427.85) identifies the presence of two clusters characterized by spherical distribution and equal volume. The “Low Severity” (LOW) cluster (51% of the sample) was characterized by the presence of mean subclinical scores for both internalizing (mean 51.0 ± 6.91) and externalizing behaviors (mean 47.1 ± 6.51), whereas the “High Severity” (HIGH) cluster (49% of the sample) was characterized by high mean behavioral problems in both domains (mean: internalizing 63.7 ± 5.97; externalizing 58.9 ± 7.10). To test the distributional assumptions, we examined the distribution of methylation percentages within each cluster. Shapiro–Wilk tests were conducted separately for the LOW and HIGH severity clusters. The results showed that the distribution of methylation values in both clusters did not deviate significantly from normality (LOW: W = 0.982, *p* = 0.21; HIGH: W = 0.978, *p* = 0.17).

### 3.2. Unsupervised Machine Learning Results: Results of Cluster Analysis on Demographics Measures, Environmental Factors, and Methylation Levels

The two clusters did not differ in terms of age, sex, or SES (Table 1).

Regarding environmental factors, the HIGH cluster had significantly higher perinatal risk factors compared to the LOW cluster (χ^2^ = 7.51, *p* = 0.006 coefficient Phi = 0.20). Compared to the HIGH cluster, the LOW cluster had higher methylation levels in *BDNF* CpGs 4 (t = 2.19, *p* = 0.030, Cohen’s d = 0.03) and 5 (t = −2.90, *p* = 0.004, Cohen’s d = −0.41), *IGF2* CpG 2 (t = −2.14, *p* = 0.034, Cohen’s d = −0.30), and *OXTR_PR* CpG 5 (t = −2.01, *p* = 0.044, Cohen’s d = −0.29). No other significant differences were found (Table 1).

## 4. Discussion

The present study used an unbiased, data-driven approach to divide a sample of adolescents initially referred for internalizing/externalizing problems in childhood into two subgroups based on their CBCL profiles for Internalizing and Externalizing [8].

The results of the best model showed the presence of two independent clusters, namely LOW and HIGH. Participants belonging to the LOW cluster had CBCL/6-18 scores in the typical range, whereas subjects in the HIGH cluster had higher externalizing scores associated with internalizing difficulties in the borderline range of clinical severity. Approximately half of the help-seeking children in our sample belonged to the HIGH cluster during adolescence. Adolescence is a very sensitive period of life in which challenging developmental changes often intermingle with psychopathological traits, especially when risk factors are present. Previous studies by our research group [45,54] have shown that the presence of a profile characterized by simultaneous internalizing and externalizing problems significantly impairs an individual’s emotional and behavioral functioning, leading to the so-called emotional and behavioral dysregulation [55].

Two previous studies conducted in an Italian setting [5,7] used cluster analysis to identify different subgroups of participants in two different community-based samples of adolescents. Both papers found a four-cluster solution for the division of the entire cohort of participants. Although they focused on different psychopathological domains, approximately 25–30% of the adolescents in the studies by Amendola et al. [7] and Muratori et al. [5] had significantly high levels of psychopathology. The discrepancies in the number and psychopathological characteristics of the clusters between these previous studies and the current study could be due to significant methodological differences, such as the sample sizes of the studies, the clustering methods, and the sample itself (community-based vs. clinically referred samples).

Having identified the two subgroups, we next found that the two clusters differed in terms of their exposure to environmental risk factors, particularly perinatal complications, and methylation of *BDNF*-, *IGF2*-, and *OXTR*-specific CpGs.

Conversely, it is important to note that the two clusters were similar in terms of sex, age, and SES. These factors are considered in the literature as potential confounders that are often associated with differences in the expression of psychopathology [5,56].

Recent studies have investigated the complex relationships between early environmental events, biological and epigenetic profiles, and the presence of psychopathological symptoms [57].

In line with the literature [13,58], our results regarding cluster characterization suggest a stronger presence of pre/perinatal risk factors in the HIGH cluster compared to the LOW cluster. Few studies focused on the presence of psychopathological problems in adolescence or later life, but previous findings suggest that perinatal events may influence biological stress and methylation mechanisms. These changes in turn represent potential risk factors for the development of internalizing and externalizing traits.

*BDNF*, the most abundant neurotrophin, plays a critical role in the regulation of neurogenesis and neurodevelopment, synaptic plasticity, and connectivity throughout life [59]. Previous studies have shown that *BDNF* is involved in dopaminergic, cholinergic, and serotonergic regulation, with an association between altered methylation profiles and the occurrence of internalizing and externalizing symptoms [60]. Our results showed a lower percentage of methylation in two CpGs of *BDNF* intron 1 in the HIGH subgroup compared to the LOW subgroup. Most studies investigating BDNF methylation in relation to the presence of psychopathology found an increase in DNA methylation [24,60]. In particular, Kundakovic and colleagues [31] found an increase in methylation levels in this region in peripheral tissues of both animal and human models after exposure to toxic substances during pregnancy. In addition, a change in methylation in intron 1 due to stress during pregnancy was found in mothers and newborns [32,33]. Otherwise, some evidence from animal and human studies suggests that the effects of life stressors and various confounding factors lead to mixed results, with both an increase and decrease in the expression of the gene being associated with various psychiatric disorders [61]

*IGF2* is abundantly expressed during pregnancy and in the brain, and alterations in its pathways have been associated with fetal and infant growth and with exposure to risk factors before birth [26,62,63,64]. Our results showed a lower percentage of methylation in IGF2 CpG2 in the HIGH compared to the LOW cluster. Although the literature is still at an early stage in interpreting the association between the methylation of *IGF2* and increased risk of psychopathological symptoms and disorders [27], our results are consistent with previous studies that found a specific association between increased anxiety and decreased methylation of CpG5 of the *IGF2* DMR [64].

Oxytocin has been found to play a crucial role in parturition and lactation as well as affiliative/prosocial behavior and consequently social skills and cognition related to empathy [65]. For these reasons, alterations in the methylation of *OXTR* have been associated with autistic spectrum disorders, callous–unemotional traits, and depression [66,67,68,69]. In the *OXTR_PR*, we found a lower percentage of methylation in one of the analyzed CpGs in the HIGH group. Again, the literature, mainly focusing on adult samples, seems to be heterogeneous, with results pointing in the opposite direction. However, other studies are consistent with our findings, suggesting that reduced methylation of OXTR is associated with higher psychopathology [70]

As is consistent with previous findings [71,72], our present results suggest that adolescence is a sensitive age for the study of DNA methylation in relation to psychopathology. However, direct comparisons with previous literature are limited by the considerable heterogeneity in terms of analytical methods. Specifically, in our opinion, heterogeneity in this field is caused by the following three main issues: (i) Differences in methods used to evaluate all variables involved: different studies which were interested in the same four candidate genes (i.e., *BDNF*, *FKBP5*, *IGF2*, and *OXTR*) took into consideration different segments of the genes and used different methodologies in different biological tissues, in pre- and post-mortem samples to evaluate their methylation. Moreover, regarding behavioral problem measures, we took the variables as continuous; the majority of studies compared groups of patients with or without psychiatric disorders. (ii) DNA methylation varies over an individual’s lifespan and it shows some peculiarities during adolescence [65]; studies during this developmental period are more scarce compared to those conducted in early childhood and later adulthood. (iii) We could not take into account possible confounding factors regarding methylation (e.g., genotyping [61,72]).

The present study suffered from several limitations. Firstly, the results are limited to our sample due to the size and heterogeneity of the sample analyzed (e.g., in terms of clinical diagnoses in childhood). Furthermore, the stability and reproducibility of the present two-cluster solution were not tested on another group of participants. Second, the cross-sectional nature of this work is a limitation when examining associations with factors such as methylation that are known to vary with age. Future extensions of this work should utilize a longitudinal design to disentangle the relationships between environment and epigenetic factors in relation to predisposition to psychopathological traits. Furthermore, pre- and perinatal risks and SLE data were collected retrospectively at the second wave of data collection and could therefore be biased by the different levels of parents’ memories.

Although the study of DNA methylation from saliva samples shows a different composition of cell types, there is evidence that they are a better choice for the study of DNA methylation in psychiatric disorders compared to blood samples [73]. In fact, saliva reflects methylation levels in the brain to a greater extent than blood [74]. Moreover, saliva sampling is a much more convenient, non-invasive, and safe method for biomarker testing, especially in studies recruiting large samples of patients with psychopathology [73,75].

In addition, the univariate nature of the comparisons between clusters with respect to the environmental and biological predictors under consideration limits our understanding of any interactions between these factors, which should be further investigated in future studies. Another potential limitation is that it was not possible to examine the relationship between childhood diagnoses and the presence of psychopathology during adolescence, nor with parental psychopathology or parenting style.

Furthermore, despite their essential role in psychiatric research, candidate gene studies frequently lack reproducibility. This is primarily attributed to limitations such as small sample sizes or the occurrence of false-positive results [76].

## 5. Conclusions

In this paper, two subgroups of adolescents were identified in a clinically referred sample of children who were characterized by relatively “high” and “low” levels of psychopathology, regardless of their categorical clinical diagnosis. Participants in the HIGH cluster were more likely to experience perinatal negative events than those in the LOW cluster. The HIGH cluster is also characterized by particular methylation patterns of *IGF2*, *BDNF*, and *OXTR*. Overall, these findings confirm the existence of a strong association between early adverse events, DNA methylation, and the presence of psychopathology in adolescence.

## Figures and Tables

**Table 1:** Characterization of the two clusters.

	Cluster LOW	Cluster HIGH	Statistical Value	*p*
N (%)	102 (51%)	98 (49%)	-	-
Age (Mean ± SD)	14.5 ± 2.02	14.4 ± 2.31	−0.59 ^a^	0.554
Sex (Male/Female)	76:26	77:21	0.45 ^b^	0.498
SES (Mean ± SD)	50.8 ± 20.3	47.0 ± 18.3	−1.37 ^a^	0.172
**Clinical variables** (Mean ± SD)				
Internalizing	51.0 ± 6.91	63.7 ± 5.97	13.97 ^a^	**<0.001**
Externalizing	47.1 ± 6.51	58.9 ± 7.10	12.24 ^a^	**<0.001**
**Environmental risk**				
SLEs	Present: 37%	Present: 48%	1.93 ^b^	0.165
Perinatal risk factors	Present: 46%	Present: 66%	7.51 ^b^	**0.006**
**Methylation level** (%) (Mean ± SD)				
*BDNF* CpG1	1.71 ± 0.51	1.65 ± 0.44	−0.81 ^a^	0.420
*BDNF* CpG2	0.40 ± 0.32	0.41 ± 0.18	0.14 ^a^	0.889
*BDNF* CpG3	0.40 ± 0.20	0.42 ± 0.17	−0.76 ^a^	0.449
*BDNF* CpG4	0.33 ± 0.14	0.28 ± 0.17	2.19 ^a^	**0.030**
*BDNF* CpG5	0.54 ± 0.25	0.46 ± 0.16	−2.90 ^a^	**0.004**
*BDNF* CpG6	0.47 ± 0.17	0.46 ± 0.21	−0.35 ^a^	0.727
*BDNF* CpG7	0.53 ± 0.41	0.46 ± 0.18	−1.52 ^a^	0.131
*BDNF* CpG8	0.79 ± 0.36	0.77 ± 0.25	−0.47 ^a^	0.640
*BDNF* CpG9	0.63 ± 0.27	0.65 ± 0.27	0.59 ^a^	0.560
*BDNF* CpG10	0.83 ± 0.29	0.83 ± 0.29	−0.07 ^a^	0.944
*BDNF* CpG11	0.59 ± 0.19	0.51 ± 0.15	−0.45 ^a^	0.660
*FKBP5* CpG1	72.38 ± 4.66	72.32 ± 5.59	−0.08 ^a^	0.935
*FKBP5* CpG2	92.87 ± 3.81	92.76 ± 3.95	−0.20 ^a^	0.844
*FKBP5* CpG3	90.22 ± 4.59	89.89 ± 5.03	−0.51 ^a^	0.614
*IGF2* CpG1	49.58 ± 5.73	48.95 ± 5.04	−0.83 ^a^	0.409
*IGF2* CpG2	39.05 ± 3.86	37.65 ± 5.29	−2.14 ^a^	**0.034**
*IGF2* CpG3	40.42 ± 4.60	39.47 ± 4.67	−1.44 ^a^	0.152
*IGF2* CpG4	34.61 ± 4.92	33.48 ± 5.16	−1.58 ^a^	0.115
*IGF2* CpG5	0.08 ± 0.05	0.08 ± 0.05	0.20 ^a^	0.840
*OXTR_E3* CpG1	7.94 ± 3.38	7.93 ± 3.10	−0.03 ^a^	0.980
*OXTR_E3* CpG2	6.56 ± 3.01	6.44 ± 2.87	−0.31 ^a^	0.759
*OXTR_E3* CpG3	4.65 ± 2.59	4.70 ± 2.14	0.15 ^a^	0.884
*OXTR_E3* CpG4	4.48 ± 2.83	4.56 ± 2.42	0.22 ^a^	0.826
*OXTR_E3* CpG5	2.01 ± 1.42	1.98 ± 1.19	−0.20 ^a^	0.840
*OXTR_E3* CpG6	8.30 ± 3.72	8.49 ± 3.98	0.33 ^a^	0.747
*OXTR_E3* CpG7	5.86 ± 3.12	5.93 ± 2.96	0.16 ^a^	0.872
*OXTR_E3* CpG8	2.95 ± 1.93	3.12 ± 1.89	0.62 ^a^	0.535
*OXTR_E3* CpG9	5.24 ± 3.02	5.40 ± 2.85	0.40 ^a^	0.690
*OXTR_E3* CpG10	5.80 ± 3.16	5.81 ± 2.92	0.02 ^a^	0.981
*OXTR_E3* CpG11	5.67 ± 3.51	5.84 ± 3.34	0.36 ^a^	0.718
*OXTR_E3* CpG12	3.04 ± 2.24	3.09 ± 2.01	0.15 ^a^	0.877
*OXTR_E3* CpG13	4.82 ± 3.43	5.10 ± 3.15	0.58 ^a^	0.558
*OXTR_E3* CpG14	3.02 ± 2.09	2.93 ± 1.98	−0.30 ^a^	0.764
*OXTR_E3* CpG15	4.26 ± 2.51	3.40 ± 2.33	−0.77 ^a^	0.440
*OXTR_I1* CpG1	1.76 ± 0.73	1.69 ± 0.81	−0.69 ^a^	0.492
*OXTR_I1* CpG2	2.76 ± 0.94	2.73 ± 1.10	−0.23 ^a^	0.822
*OXTR_I1* CpG3	7.36 ± 2.04	7.15 ± 2.09	−0.71 ^a^	0.476
*OXTR_I1* CpG4	2.92 ± 1.71	2.75 ± 1.06	−0.81 ^a^	0.417
*OXTR_I1* CpG5	36.33 ± 4.87	35.89 ± 4.97	−0.64 ^a^	0.522
*OXTR_I1* CpG6	38.70 ± 5.60	38.29 ± 5.58	−0.53 ^a^	0.597
*OXTR_I1* CpG7	62.71 ± 4.91	61.45 ± 5.17	−1.77 ^a^	0.078
*OXTR_I1* CpG8	43.77 ± 4.57	43.25 ± 5.24	−0.76 ^a^	0.446
*OXTR_I1* CpG9	23.43 ± 5.00	23.32 ± 4.79	−0.16 ^a^	0.868
*OXTR_I1* CpG10	8.55 ± 2.58	7.97 ± 2.25	−1.68 ^a^	0.093
*OXTR_I1* CpG11	10.92 ± 2.99	10.44 ± 3.00	−1.14 ^a^	0.257
*OXTR_I1* CpG12	12.37 ± 3.97	11.77 ± 3.15	−1.17 ^a^	0.243
*OXTR_I1* CpG13	13.40 ± 3.45	13.03 ± 3.26	−0.79 ^a^	0.430
*OXTR_PR* CpG1	91.63 ± 2.29	91.05 ± 2.20	−1.80 ^a^	0.072
*OXTR_PR* CpG2	78.71 ± 4.09	78.39 ± 4.68	−0.50 ^a^	0.615
*OXTR_PR* CpG3	79.64 ± 4.13	78.87 ± 3.94	−1.36 ^a^	0.175
*OXTR_PR* CpG4	64.66 ± 5.94	63.93 ± 4.64	−0.97 ^a^	0.335
*OXTR_PR* CpG5	85.13 ± 2.81	84.34 ± 2.65	−2.01 ^a^	**0.044**
*OXTR_PR* CpG6	47.53 ± 5.07	46.42 ± 4.96	−1.57 ^a^	0.118
*OXTR_PR* CpG7	71.09 ± 4.92	70.19 ± 3.78	−1.46 ^a^	0.147

^a^ = Bonferroni-adjusted *t*-test; ^b^ = χ^2^; SD = standard deviation; SES = socioeconomic status; *BDNF* = *Brain-derived neurotrophic factor*, intron 1 chr11:27723077–27723244, 11 CpGs; *FKBP5* = *FK506-binding protein 5*, intron 7 chr6:35558405–35558550, 3 CpGs; *IGF2* = *Insulin-like growth factor-2*, differentially methylated region DMR, chr11:2169373–2169658, 5 CpGs; *OXTR_E3* = *Oxytocin receptor*, promoter, chr3:8811488–8811837, 7 of 9 CpGs; *OXTR_I1* = *Oxytocin receptor*, intron 1, chr3:8810654–8810919, 13 CpGs; *OXTR_PR* = *Oxytocin receptor*, exon 3, chr3:8809340–8809530, 15 CpGs. Bold value indicates significant contrasts.

**Figure A1:**
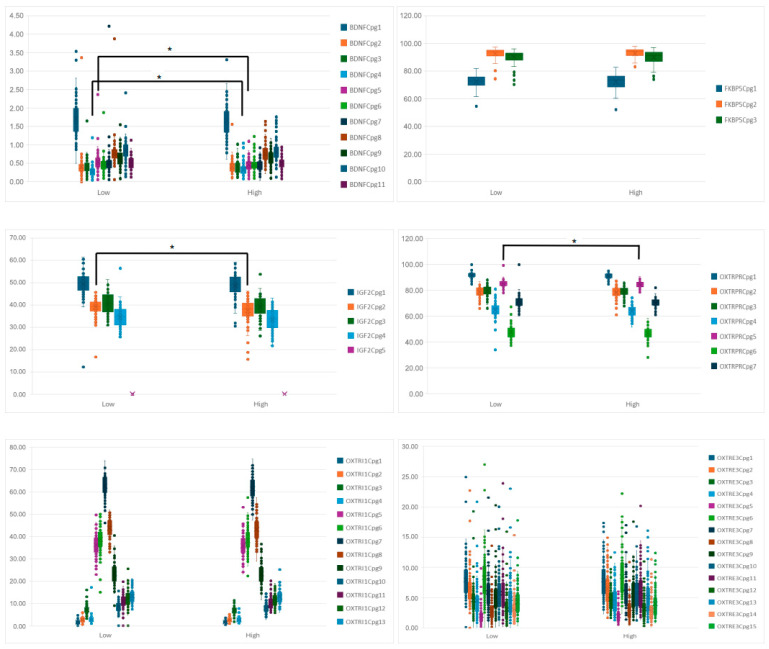
Boxplots showing the distribution of percentage methylation for each of the CpGs analyzed; significant differences between clusters in methylation percentages are marked with asterisks. Notes: For each image, the *x*-axis represents the LOW and HIGH clusters; the *y*-axis represents the methylation percentage.

## Data Availability

The datasets used and/or analyzed during the current study are available from the corresponding author on reasonable request.

## Abbreviations

The following abbreviations are used in this manuscript:

CBCL	Child Behavior Checklist/6-18
*BDNF*	*Brain-derived neurotrophic factor*
*FKBP5*	*FK506-binding protein 5*
*IGF2*	*Insulin-like growth factor-2*
*OXTR*	*Oxytocin receptor*
*OXTR_PR*	*Oxytocin receptor* promoter
ASEBA	Achenbach System of Empirically Based Assessment
SLEs	Stressful life events
DAWBA	Development and Well-Being Assessment
FMM	Finite mixture model
BIC	Bayesian information criterion

## Appendix A. Methylation Protocol

The methylation status of specific portions of the *BDNF*, *FKBP5*, *IGF2*, and *OXTR* genes was assessed by PCR amplification of bisulfite-treated DNA followed by next-generation sequencing (NGS). We analyzed *IGF2* differentially methylated region (DMR) (chr11:2169373–2169658, 5 CpGs); *BDNF* intron 1 (chr11:27723077–27723244, 11 CpGs); three regions of *OXTR*: promoter (chr3:8811488–8811837, 7 of 9 CpGs analyzed), intron 1 (chr3:8810654–8810919, 13 CpGs), and exon 3 (chr3:8809340–8809530, 15 CpGs); FKBP5 intron 7 (chr6:35558405–35558550, 3 CpGs).

Methylation levels were determined in DNA from saliva using bisulfite modification followed by NGS. The methylation analysis was performed in 2020, while saliva samples were collected from 2010 to 2013. Saliva samples for epigenetic analysis were obtained either by placing a salivary swab in the child’s mouth (for younger children) or by asking the child to spit into a tube (for older children). The saliva collection procedure was performed non-invasively, using the ORAcollect OC-175 kits (DNA Genotek, Ottawa, ON, Canada). After collection, each sample was stored at 4 °C and genomic DNA was extracted a maximum of 72 h later, following manufacturer’s protocols and quantified on a Qubit 2.0 fluorometer (Invitrogen, Life Technologies, Carlsbad, CA, USA). DNA samples were then stored at −20. We assessed DNA purity by measuring 260/280 and 260/230 absorbance ratios on a NanoDrop spectrophotometer (Thermo Fisher Scientific, Waltham, MA, USA). Bisulfite conversion was performed on 200 ng of genomic DNA using the EZ DNA methylation lightning kit (ZymoResearch, Inc, Irvine, CA, USA). Primers were designed using Bisulfite Primer Seeker (http://www.zymoresearch.com/tools/bisulfite-primer-seeker, accessed on 5 August 2022). Specific tails were added to the primers in order to allow synthesis of SureSelect-style libraries of methylated fragments. Gene-specific PCR amplifications were performed on 20 ng of bisulfite-treated DNA using Taq Gold (Life Technologies, Carlsbad, CA, USA). Cycling consisted of 5 min pre-activation at 95 °C, followed by 35 cycles of 94 °C denaturation for 15 s, 58 °C annealing for 20 s (56 °C for FKBP_i7, 55 °C for OXTR_E3), and 72 °C elongation for 1 min 30 s. All PCR products were tested on 2% agarose TAE gels, treated with Ilustra Exo Pro-STAR (GE Healthcare; Dublin, Ireland) to eliminate unincorporated primers, and quantified on a Bioanalyzer 2100 (Agilent; Santa Clara, CA, USA).

Secondary PCR was conducted by amplifying pooled equimolar amounts of all gene-specific PCRs from each subject with Agilent Adapters1+2 using Herculase 1 (Agilent; Santa Clara, CA, USA). Cycling consisted of 2 min pre-activation at 68 °C, followed by 8 cycles of 98 °C denaturation for 30 s, 58 °C annealing for 30 s, and 72 °C elongation for 1 min. Products were again purified with Ilustra Exo Pro-STAR and 4 mL aliquots of each sample were amplified with a SureSelect Custom Amplicon Index Kit (Agilent; Santa Clara, CA, USA) containing eight forward (i5) and twelve reverse (i7) index primers, allowing unique tagging of up to 96 samples. Cycling consisted of 2 min pre-activation at 98 °C, followed by 14 cycles of 98 °C denaturation for 30 s, 58 °C annealing for 30 s, and 72 °C elongation for 1 min. Again all PCR products were checked on a 2% agarose TAE gel, purified with AMPure XP beads (Beckman Coulter; Brea, CA, USA), quantified on a Bioanalyzer 2100, then approximately equimolar aliquots of each product were pooled in the final library, containing six specific DNA fragments from up to 96 subjects. The library was sequenced on a NEXTSeq 500 (Illumina Inc., San Diego, CA, USA) using a v2 Reagent kit, 300 cycles PE.

Paired-end reads from each sample were independently aligned to all the reference sequences by a parallel striped Smith–Waterman algorithm. Only paired reads that aligned coherently to the same reference sequence were retained. At each CpG site in each sequence, the four base frequencies were evaluated and reported along with the C→T percentage.

## Appendix B

**Table A1:** Distribution of methylation: range, mean, and standard deviation of individual CpG units within BDNF, FKBP5, IGF2, and OXTR regions.

Methylation Level	Chromosomal Positions (GRCh37/hg19)	Min (%)	Max (%)	Mean (%)	SD (%)
*BDNF* CpG1	chr11: 27723218–27723219	0.50	3.54	1.68	0.47
*BDNF* CpG2	chr11: 27723214–27723215	0.00	3.37	0.41	0.27
*BDNF* CpG3	chr11: 27723203–27723204	0.03	1.65	0.41	0.19
*BDNF* CpG4	chr11: 27723190–27723191	0.05	1.20	0.30	0.16
*BDNF* CpG5	chr11: 27723161–27723162	0.06	2.37	0.50	0.22
*BDNF* CpG6	chr11: 27723159–27723160	0.08	1.88	0.46	0.19
*BDNF* CpG7	chr11: 27723143–27723144	0.04	4.22	0.49	0.32
*BDNF* CpG8	chr11: 27723137–27723138	0.06	3.88	0.78	0.32
*BDNF* CpG9	chr11: 27723128–27723129	0.08	1.55	0.64	0.24
*BDNF* CpG10	chr11: 27723125–27723126	0.13	2.41	0.83	0.29
*BDNF* CpG11	chr11: 27723095–27723096	0.09	1.20	0.51	0.18
*FKBP5* CpG1	chr6: 35558438–35558439	52.32	82.94	72.35	5.12
*FKBP5* CpG2	chr6: 35558488–35558489	74.46	98.11	92.82	3.87
*FKBP5* CpG3	chr6: 35558513–35558514	70.39	97.21	90.05	4.80
*IGF2* CpG1	chr11: 2169400–2169401	12.27	61.46	49.27	5.40
*IGF2* CpG2	chr11: 2169499–2169500	15.75	46.28	38.37	4.66
*IGF2* CpG3	chr11: 2169515–2169516	26.15	53.86	39.95	4.65
*IGF2* CpG4	chr11: 2169518–2169519	21.74	56.50	34.06	5.06
*IGF2* CpG5	chr11: 2169577–2169578	0.00	0.25	0.08	0.05
*OXTR_E3* CpG1	chr3: 8809364–8809365	0.11	24.93	7.94	3.23
*OXTR_E3* CpG2	chr3: 8809367–8809368	0.00	22.68	6.50	2.94
*OXTR_E3* CpG3	chr3: 8809369–8809370	0.11	19.25	4.68	2.38
*OXTR_E3* CpG4	chr3: 8809387–8809388	0.00	20.84	4.52	2.63
*OXTR_E3* CpG5	chr3: 8809394–8809395	0.06	9.84	1.99	1.31
*OXTR_E3* CpG6	chr3: 8809399–8809400	0.12	26.99	8.39	3.85
*OXTR_E3* CpG7	chr3: 8809413–8809414	0.00	21.53	5.90	3.04
*OXTR_E3* CpG8	chr3: 8809417–8809418	0.09	13.58	3.04	1.91
*OXTR_E3* CpG9	chr3: 8809422–8809423	0.12	20.26	5.32	2.93
*OXTR_E3* CpG10	chr3: 8809425–8809426	0.33	20.02	5.80	3.04
*OXTR_E3* CpG11	chr3: 8809428–8809429	0.24	23.89	5.75	3.42
*OXTR_E3* CpG12	chr3: 8809433–8809434	0.03	15.91	3.07	2.13
*OXTR_E3* CpG13	chr3: 8809437–8809438	0.06	23.02	4.96	3.29
*OXTRE_3* CpG14	chr3: 8809442–8809443	0.18	15.33	2.98	2.04
*OXTR_E3* CpG15	chr3: 8809464–8809465	0.32	17.75	4.13	2.42
*OXTR_I1* CpG1	chr3: 8810889–8810890	0.06	4.80	1.73	0.77
*OXTR_I1* CpG2	chr3: 8810874–8810875	0.06	6.71	2.75	1.02
*OXTR_I1* CpG3	chr3: 8810862–8810863	1.95	16.12	7.26	2.07
*OXTR_I1* CpG4	chr3: 8810855–8810856	0.84	17.28	2.84	1.43
*OXTR_I1* CpG5	chr3: 8810832–8810833	23.02	53.09	36.12	4.91
*OXTR_I1* CpG6	chr3: 8810807–8810808	15.10	57.52	38.50	5.58
*OXTR_I1* CpG7	chr3: 8810797–8810798	46.11	74.84	62.09	5.07
*OXTR_I1* CpG8	chr3: 8810774–8810775	28.89	57.48	43.52	4.90
*OXTR_I1* CpG9	chr3: 8810733–8810734	9.03	40.46	23.38	4.88
*OXTR_I1* CpG10	chr3: 8810708–8810709	0.07	15.68	8.27	2.44
*OXTR_I1* CpG11	chr3: 8810699–8810700	0.17	20.24	10.68	3.00
*OXTR_I1* CpG12	chr3: 8810681–8810682	0.07	25.62	12.08	3.60
*OXTR_I1* CpG13	chr3: 8810679–8810680	6.10	25.23	13.22	3.36
*OXTR_PR* CpG1	chr3: 8811763–8811764	84.80	99.82	91.35	2.26
*OXTR_PR* CpG2	chr3: 8811758–8811759	61.12	87.95	78.55	4.38
*OXTR_PR* CpG3	chr3: 8811756–8811757	66.11	88.19	79.26	4.05
*OXTR_PR* CpG4	chr3: 8811739–8811740	33.89	80.86	64.30	5.34
*OXTR_PR* CpG5	chr3: 8811728–8811729	77.54	99.08	84.74	2.76
*OXTR_PR* CpG6	chr3: 8811601–8811602	28.20	67.18	46.98	5.04

*BDNF* = *Brain-derived neurotrophic factor*, intron 1 chr11:27723077–27723244, 11 CpGs; *FKBP5* = *FK506-binding protein 5*, intron 7 chr6:35558405–35558550, 3 CpGs; *IGF2* = *Insulin-like growth factor-2*, differentially methylated region DMR, chr11:2169373–2169658, 5 CpGs; *OXTRE_3* = *Oxytocin receptor*, promoter, chr3:8811488–8811837, 7 of 9 CpGs; *OXTR_I1* = Oxytocin receptor, intron 1, chr3:8810654–8810919, 13 CpGs; *OXTR_PR* = Oxytocin receptor, exon 3, chr3:8809340–8809530, 15 CpGs.

**Table A2:** Best model selection: Bayesian Information Criterion (BIC) values.

Number of Clusters	Spherical, Equal Volume Clusters (EII)	Spherical, Unequal Volume Clusters (VII)
1	−2910.112	−2910.112
2	**−2887.488**	**−2892.799**
3	**−2889.127**	−2898.691
4	−2902.274	−2916.547
5	−2918.169	−2933.301
6	−2932.867	−2951.756
7	−2929.585	−2947.264
8	−2941.88	−2982.887
9	−2966.898	−3001.052

Bold value indicates the top three models identified by the cluster analysis.
